# App development in a sports science setting: A systematic review and lessons learned from an exemplary setting to generate recommendations for the app development process

**DOI:** 10.3389/fspor.2022.1012239

**Published:** 2023-01-04

**Authors:** Bettina Barisch-Fritz, Claudio R. Nigg, Marc Barisch, Alexander Woll

**Affiliations:** ^1^ Institute of Sports and Sports Science, Karlsruhe Institute of Technology, Karlsruhe, Germany; ^2^ Institute of Sports Science, University of Bern, Bern, Switzerland

**Keywords:** mobile applications, checklist, barriers of implementing apps, software engineering, digitalization, digital health

## Abstract

The digital health sector is rapidly growing. With only 4% of publishers out of academic settings, it is under-represented in app development. The objective of this study is to assess the current state of app development with a systematic review and a survey within an exemplary academic setting along the following research questions: (Q1) Are software engineering principles sufficiently known in the sports science app development context? (Q2) Is the role of sports scientists in the context of app development sufficiently understood? The systematic review was conducted by two independent reviewers within databases Pubmed, Scopus, Web of Science, and IEEE Xplore. The PICO schema was used to identify the search term. We subtracted information about five main topics: development process, functional requirements and features, security, technology, and dissemination. The survey was developed by a multidisciplinary team and focused on five main topics. Out of 701 matches, 21 were included in the review. The development process was only described in seven studies. Functional requirements and features were considered in 11 studies, security in 3, technology in 13, and dissemination in 12 with varying details. Twelve respondents [mean age 33(7) years, 58% women] replied to the survey. The survey revealed limited knowledge in realization of security measures, underlying technology and source code management, and dissemination. Respondents were able to provide input on development processes as well as functional requirements and features. The involvement of domain experts is given in seven review studies and described in two more. In 50% of survey respondents, the role in app development is defined as a research assistant. We conclude that there is a varying degree of software engineering knowledge in the sports science app development context (Q1). Furthermore, we found that the role of sports scientists within app development is not sufficiently defined (Q2). We present recommendations for improving the success probability and sustainability of app development and give orientation on the potential roles of sports scientists as domain experts. Future research should focus on the generalizability of these findings and the reporting of the app development process.

## 1. Introduction

Mobile applications (apps) running on smartphones and tablets have substantially influenced how we communicate, consume, conduct daily tasks, and organize our lives (1). The introduction of Apple's iPhone in 2007 initiated and enabled the enormous spread of apps. The development of apps for mobile devices is one of the fastest-growing industry sectors, with the expected revenue tripling from 2018 (365 billion US dollars) to 2023 (935 billion US dollars) (2). In 2018, more than 105 billion apps were downloaded (3). In 2021, iOS and Android users could choose between 5.5 million apps (2). Out of these developments, it can be stated that almost all companies have realized that mobile apps are a necessity to create customer value and retain/increase their revenue (4).

A significant share of this overall market is the digital health sector. This sector is expected to grow by 11.8% yearly from 11.9 billion US dollars in 2022 (5). In total, more than 325,000 digital health apps are available, with a daily growth rate of 200 new apps (6, 7). Digital health solutions can be grouped into (1) solutions that improve system efficiency but with no measurable patient outcome benefit; (2) solutions that inform or deliver basic monitoring and encourage behavior change and self-management; and (3) clinical decision support and prediction models that guide treatment, deliver active monitoring, calculate, and/or diagnose (8). Considering apps used in the context of health, fitness, and sports, the spectrum is also broad, including physical performance, physical activity promotion, training, rehabilitation, exergaming, and diagnostics.

In 2017, nonhealthcare companies made up 23% of the digital health market share and only 4% were out of an academic setting (6). This trend has been strengthened, and the main publishers of digital health apps are start-up companies (9). The low number of publishers out of universities most likely does not cover the number of scientists focusing on app development. We assume that many developed digital health apps out of academia do not enter the market. Guo et al. stated that a growing number of academic centers have developed and evaluated digital health apps. However, the focus on high-impact publications and thus time-consuming studies delay or even ruin the implementation of potentially valuable solutions (8). This might be in line with findings that more than 95% of available apps have not been scientifically tested (10). For example, Larsen et al. (11) found only one app that included a reference to published literature in the case of mental health apps.

Apart from that, the attractiveness of the German digital health market, with 90% of the population enrolled in public health insurance, is very high (12). It is ranked second after the United States (9). The Digital Healthcare Act (DVG, November 7, 2019) was passed to support healthcare innovation and digitalization (13). It also provides opportunities for developments outside of academia to support evidence-based research. However, there are several barriers to digital health implementation (12, 14, 15). Several studies identified financial, legal, social, and ethical barriers to implementation and security concerns (16). Challenges in the digital health app market are complex, and often country-specific regulations, like Good Manufacturing Practice (9), security requirements for handling sensitive personally identifiable data, and reimbursement issues, exist (17).

The success of a digital solution is quite difficult to analyze. Objectively, the annual download numbers are used as a measure. These numbers depend on several factors that are strongly influenced by the experience of the publisher (6) and the available development/marketing budget. This holds in particular for apps with revenue above US$1M. Apart from that, when people have downloaded an app, more than 75% will not open it again (18). This represents what users expect (fast loading time, ease of use, and delight during interaction) and how convincing an app must be at the first interaction.

Llorens-Vernet and Miró (19) identified in their study a set of criteria for digital health-related apps. Out of published studies, guidelines, and standards, they identified the categories of usability, privacy, security, appropriateness and suitability, transparency and content safety, technical support and updates, and technology. With this set of criteria, they help care providers, developers, patients, and other stakeholders to guide the development of health-related apps and, potentially, measure their quality (19). To realize these criteria, basic knowledge of software engineering principles and the corresponding software development process is necessary. Moreover, domain experts, also known as subject matter experts, need to be involved during the entire development process (20).

From an academic perspective, the development of digital health solutions is challenging in a rapidly changing technology landscape (17). The above-mentioned barriers and the cross-disciplinary nature of app development are not well evaluated. No research is available examining the development process in academic settings from the domain expert view. Therefore, a closer look at the development processes at universities and potential success criteria for digital health app development is required.

The aim of this study is to give an overview of the app development process within an academic sports science setting and analyze how sports scientists as domain experts are involved in the app development process. We started a systematic review to examine the current state of app development in the digital health, fitness, and sports field with a focus on physical activity applications. Furthermore, we exemplarily observed the view of sports scientists through a survey. The following research questions were formulated:

Q1: Are software engineering principles sufficiently known in the sports science app development context?

Q2: Is the role of sports scientists in the context of app development sufficiently understood?

With the examination of the current state of app development by a systematic literature review and a survey within an exemplary setting, we further aimed to derive recommendations for structuring the app development process in academic sports and fitness and health settings.

## 2. Materials and methods

### 2.1. Systematic review

A systematic literature review was conducted in October 2022 by two independent reviewers (BB-F, MB) using the following electronic databases: Pubmed, Scopus, Web of Science, and IEEE Xplore. The PICO schema (21) was used to identify the inclusion criteria: The population in the studies should be out of the area of digital health. As an intervention, they should report about apps out of the field of sports science addressing the topic of physical activity, training, or exercise. For term comparison, information on the app development process must be given. The outcome variable could not be integrated into the search term but focuses on the app development process and the involvement of domain experts, i.e., sports scientists. Additionally, records had to be published in English or German, and empirical articles of all study designs focusing on app development and also conference papers were included. The search was made from January 2012 to October 2022.

Abstract management tool Rayyan was used to identify and delete duplicates. Two authors (BB-F and MB) independently screened titles and abstracts based on the inclusion criteria (1: mHealth app development, 2: apps out of the field of sports science, physical activity, exercise, and training). The abstracts that met the inclusion criteria were retained for the full-text screening step. Disagreements regarding the inclusion of studies were discussed with the reviewers, and reasons for the exclusion of the full texts were recorded. The following information of the included studies was extracted: (1) authors and year and country of publication, (2) study aim and design, (3) information about the involvement of domain experts/role of sports scientists, (4) app development process, (5) functional requirements and features, (6) security, (7) technology, and (8) dissemination. The intention for the information about the app development process, functional requirements and features, security, technology, and dissemination is the same as for the survey and is described in Section 2.2.2.

### 2.2. Survey

#### 2.2.1. Study design and recruitment

The aim of capturing the current state of app development within the Institute of Sports and Sports Science at the KIT was addressed by a survey. The survey study aimed to create transparency in app development processes and obtain details on strengths, weaknesses, and opportunities of the current approach. An online survey was developed. The Checklist for Reporting Results of Internet E-Surveys (CHERRIES) (22) guided this project. The survey was conducted without collecting and storing personal information. No ethical approval was applied to this study, as no risks, losses, or disadvantages for the respondents could have been identified. Furthermore, no fundamental ethical principles (23) would have been violated during recruitment, data collection, and analysis.

The survey was carried out in March 2019. It was password-protected and thus only accessible to the recruited sample. The recruitment of survey respondents took place in two steps. (1) Sample selection: The sample was selected out of 65 academic staff members of the Institute of Sports and Sports Science, KIT. In collaboration with the professors, 16 persons were identified that were at that time actively involved in app development. (2) Survey invitation: An invitation explaining the survey (informed consent) was given to the 16 selected persons and afterward they received access to the online survey in which they might have voluntarily participated.

#### 2.2.2. Survey design

The survey was grouped into seven different sections, each assessing a different aspect of the app development process. The sections and the questions of the survey were developed by a multidisciplinary team consisting of experts from sports science and computer science:

• Professor in sports science: working in sports science for 25 years;
• Postdoc in sports science: working on digitalization of sports science for 3 years; and
• Software engineering professional: more than 20 years of experience in industrial projects as a developer and software architect.

The different sections of the survey are explained in the following subsections.

##### 2.2.2.1. Demographics

To describe the sample, information about age, gender, highest academic degree, the primary area of expertise, and role in app development of the respondents was assessed. The area of expertise provides us with an indication of the background of the respondents to analyze the given competencies for conducting app development projects.

##### 2.2.2.2. Project information

To understand the scope and the goals of the app development projects, we assessed the following information:

• Project title: defines the name of the app development project and allows us to check whether multiple responses for the same project have been received.
• Project description and goals: Allowed us to understand the content, scope, and goal of the project. In particular, whether the goal of the project was to answer research questions (see Section 1).
• Role within development: We wanted to understand whether the role of the respondent was clearly defined. If the respondent is not able to answer this question, we have an indication of the lack of defined processes.

##### 2.2.2.3. Development process

The goal of this section was to understand how app development takes place and assess the quality of the development process. This includes questions about whether a development process is in place to define the responsibilities including requirements engineering and defect tracking.

##### 2.2.2.4. Functional requirements and features

A central aspect of software engineering is requirements engineering. This survey section contains questions about how the requirements for app development have been defined and which functional requirements play a major role. This allows us to identify common requirements across the different app development projects and gives us indications about the quality of requirements engineering itself.

##### 2.2.2.5. Security

Many research questions in sports science deal with personally identifiable information that needs special data protection mechanisms defined by laws (e.g., EU-DSGVO). To understand the importance of security in general and with respect to given laws, we included a couple of questions to understand what the individual apps need and to which degree an understanding of the required technology is given.

##### 2.2.2.6. Technology

In this subsection, we addressed two concerns: (1) Which technology and which knowledge about the used technology of the app are available? Common technologies can help to speed up the development across different teams and help to reduce maintenance cost. (2) Which infrastructure is used to manage the app development? The answers to these questions will indicate the professionalism that is applied and also to which degree an understanding of software engineering is available within the sports science department.

##### 2.2.2.7. Dissemination

The main concern of the questions in this section was to understand how the developed apps get from development to end users, how end users are informed about the existence of the app (marketing), and whether commercial aspects (e.g., pricing) are considered.

#### 2.2.3. Survey analysis

The findings were systematically assessed by an online survey tool (SurveyMonkey, San Mateo, USA) and statistically summarized by SPSS (version 25). Results are descriptively presented by percentage frequencies. In addition, between-group differences were calculated by factorial ANOVAs. The independent variables like gender, age group, highest degree, the primary area of expertise, and role in app development were consulted to analyze the influence on relevant variables out of the five major topics (development process, functional requirements and features, security, technology, and dissemination).

## 3. Results

### 3.1. Results of the systematic review

**Figure 1:**
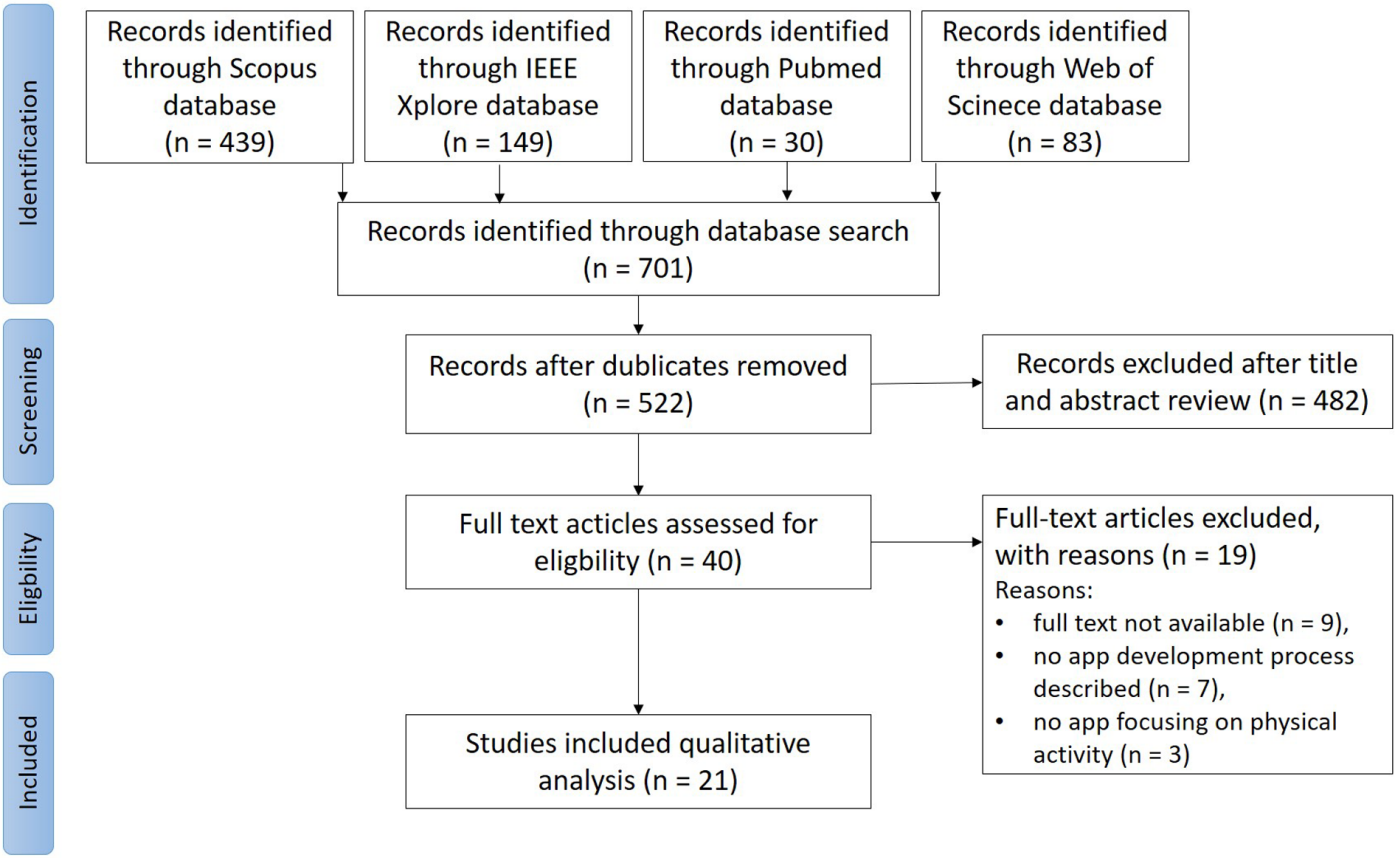
PRISMA (preferred reporting items for systematic reviews and meta-analyses) diagram.

The screening process of this systematic search is shown in Figure 1. After deleting duplicates, 522 were screened by the title and abstract. According to the inclusion criteria, 40 articles were retained in the full-text screening step. Out of this, nine were excluded as no full text was available, seven were excluded as no app was developed, and three were excluded as the reported app did not involve physical activity. The included 21 studies were qualitatively evaluated to assess how the main topics were addressed within the studies.

A summary of the findings of the 21 included studies is presented in Table 1. Nine articles were original articles, whereas 12 were conference papers. The year of publication was restricted from January 2012 to October 2022. Within these years, a maximum of three publications were found in the years 2015, 2017, 2018, and 2020. The countries from which the apps were developed are widespread. In seven articles, at least one of the authors was from an academic sports science setting. Thus, the involvement of sports scientists, i.e., domain experts, can be assumed. In two other studies, the involvement of domain experts was explicitly described. The aims of the apps can be divided into physical activity promotion for healthy and/or dedicated target groups. In two studies, the target group was not clearly described. The development process was described in seven studies, mentioned in two, and not described in twelve studies. The definition of the requirements and features was based on user experience research in five studies. The functional requirements and features were not explicitly listed in 10 studies.

**Table 1:** Findings of the systematic literature review.

Outcome	Findings (*n*)
Type of article	Original article (9) (24–32) Conference paper (12) (33–45)
Year of publication	2012 (2), 2013 (2), 2015 (3), 2016 (1), 2017 (3), 2018 (3), 2020 (3), 2021 (2), 2022 (2)
Country of publication	United States (2), Canada (2), Australia (2), Malaysia (2), Germany (1), India (1), Switzerland (1), Korea (1), United Kingdom (1), Mauritius (1), Indonesia (1), Indonesia/China (1), Taiwan (1), Poland (1), Russia (1), Thailand (1), Ireland (1)
Authors are sports scientists (at least one of the authors)	Yes (7), no (14)
Involvement of domain experts	Yes (9), no (6), no further/sufficient information (6)
Aim of the app	Physical activity promotion for dedicated/vulnerable target group (4), for healthy target group (13), for both/consideration of diseases (2), not clear/described (2)
Development process (reported app development methodology)	Detailed described (7), mentioned (2), not described (12)
Functional requirement**s** and features	Based on user experience research methods (5), not based on user experience research methods (16) Detailed requirements listed, >10 (5); mentioned requirements, <10 (6), not explicitly listed (10)
Security	Considered (3), not considered (18)
Technology	Android without details (5), Android with details (4), iOS with details (1), others (7), not mentioned (4)
Dissemination	App store (2), limited to study scope (5), not yet defined (5), no details described (9).

Consideration and measures of security were considered in three studies. The underlying technology was in nine studies on Android. Four studies described the technology in many details. Other technologies were used for seven apps, one app was based on iOS, and four studies did not mention the underlying technology. The dissemination is not detailed described in nine studies. In five studies, it was mentioned that dissemination still needs to be defined. Five developed apps were limited to study scope, and two are planned to be disseminated *via* corresponding app stores.

### 3.2. Results of the survey

The findings are collected from 12 out of 16 (75% response rate) survey responders. The reasons for the four nonresponders were not further investigated. All 12 respondents replied to the entire survey (no missing data). The results are given in percentage of the response options. The findings of the five major topics of the survey are graphically presented in the Appendix (Figures 2–6). Project information and the role of the survey respondents are also presented in Table 2.

**Table 2:** Characteristics and primary area of expertise and app project information of the respondents (*N* = 12).

Characteristic	*n* (%)
Age group in years	
26–30	5 (42)
31–35	5 (42)
36–40	1 (8)
>50	1 (8)
Gender	
Female	7 (58)
Male	5 (42)
Highest degree	
Master degree	7 (54)
Doctoral degree	3 (23)
Postdoctoral qualification	1 (8)
Others	1 (8)
Primary area of expertise^a^	
Exercise science/kinesiology	2
Health science	2
Information technology	2
Psychology	2
Physical education	1
Sociology	1
Health promotion	1
Psychophysiology	1
Project title^a^	
Smart family	6
InCoPE app	2
Sport-Zens	2
CoCa	1
Eat2beNice	1
Sportlehrer-App	1
Walking-App	1
Other	3
Role in app development	
Research assistant	6 (50)
Project manager/coordinator	3 (26)
Product owner	1 (8)
IT consultant	1 (8)
Scientific consultant	1 (8)
Who actually developed the app^a^	
App company staff	4
IT specialist	3
Project manager/coordinator	2
Research assistant	1
Project partner	1
Teacher	1

^a^ Multiple answers are possible; thus, percent values are not provided.

Eight apps were represented (some respondents were from the same projects). Half of the respondents were research assistants. External app companies and internal IT specialists were responsible for the actual development of over half of the apps. Some apps were developed by the project coordinator, research assistants, or teachers.

#### 3.2.1. Results of the main five topics

The results of the five main topics are presented in Table 3 an in the Supplementary Material. One respondent reported that they had used an established app development methodology (like SCRUM), and half of the respondents did not even know about existing methodologies. Most apps used some kind of software testing. Over half have an idea for defect management without a clear definition of responsibilities (see Table 3).

**Table 3:** Methodology used in app development (*N* = 12).

Characteristic	*n* (%)
Development methodology	
Yes, was used	1 (8)
No, was not used	5 (42)
No answer given	6 (50)
Development methodology	
Yes, SCRUM	1 (8)
No	11 (92)
Testing	
Pilot study	3 (25)
By a betaVersion	1 (8)
By the project team	1 (8)
Feasibility study	1 (8)
Not ready for testing	1 (8)
Trying it out by hand	1 (8)
No answer given	4 (33)
Defect management	
IT specialist	2 (17)
Myself	1 (8)
Everybody	1 (8)
App company	1 (8)
Project	1 (8)
Project partner	1 (8)
No answer	4 (33)
Do not know	1 (8)

The results of questions about functional requirements and features are presented in Table 4. Only one-third of the respondents conducted a stakeholder analysis to identify potential users and involved people during the development and later usage of the application. The majority of apps were developed with German language support only, which shows that the focus of the app is limited to German-speaking countries. The need for multimedia support like acoustic effects, music, short movies, and pictures was, in general, of low importance.

**Table 4:** App requirements and features (*N* = 12).

Characteristic	*n* (%)
Stakeholder analysis	
Yes	4 (36)
No	8 (64)
Importance of modern design^a^	
Somewhat important	4 (36)
Moderately important	4 (36)
Very important	3 (27)
Importance of acoustic effects^a^	
Not important at all	8 (73)
Somewhat important	2 (18)
Moderately important	1 (9)
Importance of music^a^	
Not important at all	10 (91)
Somewhat important	1 (9)
Importance of short movies^a^	
Not important at all	5 (45)
Somewhat important	3 (27)
Very important	2 (18)
Extremely important	1 (9)
Importance of pictures/graphs^a^	
Somewhat important	3 (27)
Moderately important	4 (36)
Very important	1 (9)
Extremely important	3 (27)
Language and ability to switch^b^	
German	7 (58)
German and English	3 (25)
German, English, Dutch, Spanish	1 (8)
No answer	1 (8)
Ability to switch language	1 (8)

^a^ One participant did not answer questions about the importance of several features.

^b^ Multiple answers are possible; thus, percent values are not provided.

Table 5 presents findings on security. The majority of apps do not collect personally identifiable information (PII). To comply with regulations and laws, some apps avoid the collection of PII. From a technical point of view, the respondents had limited knowledge of the realization of security measures (see Table 5) and either provided no answer (e.g., 84% in the case of storage of personally identifiable information) or did not know which security measures have been used.

**Table 5:** App security, technology, and dissemination (*N* = 12).

Characteristic	*n* (%)
Personally identifiable information (collection)	
Yes	4 (33)
No	7 (58)
No answer	1 (8)
EU_DSGVO	
Pseudonymized data storage	1 (8)
Leave out anything that could stress EU_DSGVO	1 (8)
No data over the internet	1 (8)
No answer/unable to answer	9 (75)
Storage of personally identifiable information	
Secured on an external hard drive	1 (8)
AES256 encrypted SQLite database	1 (8)
No answer given	10 (84)
Security measures	
Yes	2 (17)
No	4 (33)
Password to open app	1 (8)
Initializing process	1 (8)
No answer	3 (25)
Open source software	
Yes	5 (42)
No	5 (42)
No answer	2 (17)
Programming language/framework	
Ionic, JavaScript, Angular, CSS	1 (8)
No answer/do not know	11 (92)
Source code management	
GitHub	1 (8)
Microsoft repositories	1 (8)
No answer/do not know	10 (83)
Deployment	
Own hosting	1 (8)
iOS App Store, Google Play	1 (8)
Project homepage	1 (8)
No answer/do not know	9 (75)
Publication/marketing	
Personal contact	1 (8)
On special homepage	1 (8)
Website, Facebook, newspaper	1 (8)
Word of mouth	1 (8)
No answer	8 (67)
Pricing	
No cost	4 (33)
No answer	8 (67)
Updating	
Via App/Play Store	1 (8)
Exchange complete app	1 (8)
Advertising inside the app	1 (8)
No answer given	9 (75)

Knowledge about the underlying technology, the used frameworks, and the accessibility of the source code *via* source code management is essential to avoid costly changes in the app. Open-source software was used by about half of the apps. However, the majority of respondents did not know about the underlying app technology and how their source code was managed. The majority of respondents were unaware or unable to answer how the app shall be accessible by a user. Existing deployment infrastructure might play in future a more important role in simplifying this process. In total, 76% of the respondents gave no answers on how the app shall be published and what the corresponding marketing shall look like. In total, 33% of the apps will be free of charge. For the other apps, it is necessary to develop appropriate pricing models (see Table 5).

#### 3.2.2. Results of one-factorial ANOVA

The findings of the between-group differences are presented in Table 6. Based on the small number of respondents, a normal distribution was not given for all variables. Nonetheless, we calculated one-factorial ANOVA as the simulation study of Blanca et al. identified this statistical procedure as robust against violations (46).

**Table 6:** Results of one-factorial ANOVA.

Variables		Highest degree	Gender	Age group	Primary area of expertise	Role in app development
Development methodology	*F* (df)	*F* (3) = 0.326	*F* (1) = 0.005	*F* (3) = 0.611	*F* (4) = 3.311	*F* (3) = 0.611
	*P*	*P *= .807	*P *= .946	*P *= .627	*P *= .080	*P *= .627
Testing	*F* (df)	*F* (3) = 0.539	*F* (1) = 8.219	*F* (3) = 0.461	*F* (4) = 3.677	*F* (3) = 0.231
	*P*	*P *= .669	*P *= .017	*P *= .717	*P *= .604	*P *= .873
Stakeholder analysis	*F* (df)	*F* (3) = 0.408	*F* (1) = 0.043	*F* (3) = 0.966	*F* (4) = 1.045	*F* (3) = 0.848
	*P*	*P *= .752	*P *= .840	*P *= .460	*P *= .457	*P *= .510
Security measures	*F* (df)	*F* (3) = 0.459	*F* (1) = 0.306	*F* (3) = 3.759	*F* (4) = 1.473	*F* (3) = 1.189
	*P*	*P *= .718	*P *= .592	*P *= .060	*P *= .307	*P *= .374
Open-source software	*F* (df)	*F* (3) = 0.765	*F* (1) = 0.938	*F* (3) = 1.500	*F* (4) = 0.438	*F* (3) = 0.458
	*P*	*P *= .545	*P *= .356	*P *= .287	*P *= .779	*P *= .719
Source code management	*F* (df)	*F* (3) = 4.000	*F* (1) = 0.057	*F* (3) = 1.037	*F* (4) = 1.167	*F* (3) = 4.000
	*P*	*P *= .052	*P *= .815	*P *= .427	*P *= .402	*P *= .052
Deployment	*F* (df)	*F* (3) = 0.574	*F* (1) = 0.380	*F* (3) = 0.257	*F* (4) = 0.379	*F* (3) = 0.626
	*P*	*P *= .648	*P *= .552	*P *= .854	*P *= .817	*P *= .618
Publication/marketing	*F* (df)	*F* (3) = 4.556	*F* (1) = 1.886	*F* (3) = 2.492	*F* (4) = 0.548	*F* (3) = 1.956
	*P*	*P *= .038	*P *= .200	*P *= .134	*P *= .707	*P *= .199

The between-group differences were statistically significant for the influence of education (surveyed by the highest degree) on publication/marketing. A trend toward more experiences promoting the probability of answering specific questions on app development could be seen. Furthermore, female participants could give more often an answer on testing the app.

## 4. Discussion

This study aimed to give an overview of the current state of app development in an academic sports science setting. This aim is approached by two sources of information. The main findings of the systematic review are that app development is not comprehensively described in the articles and that no standards for reporting exist. The survey in the exemplary academic setting showed that sports scientists engaged with app development could also not give detailed information on the development processes.

### 4.1. Discussion of the five main topics

The systematic review showed that app development aiming at physical activity, fitness, or sports is described in 21 articles. Most included articles did not describe app development in detail. This point may be partly described by the finding that 12 of the included articles were conference papers. The survey among sports scientists showed that most of the respondents did not know existing development methodologies. These findings may characterize the academic setting in contrast to the free market economy where agile methodologies are dominant, especially in software development. The benefits of agile methodologies (for example, SCRUM) are well known (47).

Functional requirements and features are important factors in the development of apps focusing on physical activity. However, these were not explicitly listed in about half of the systematic review studies. The aims of the applications are mentioned in all cases; however, it is often unlear how these aims will be addressed. Furthermore, the definition of the requirements and features is given only in five studies based on user experience methods. This is astonishing as functional requirements and features are essential for the specific app, and the app’s success must be defined throughout a user-centered development process, starting among other steps with stakeholder and requirement analysis, which was already mentioned in 2001 (48). Until now, there are a large number of published methods about user experience research and also a large number of articles focusing on this issue in the app development process. Results of the survey showed that usability issues were not the prime focus. This deficit is important to take countermeasures as recommended by Michie et al. to make the development person-centered and agile as well as iterative using mixed methods to meet user requirements (17).

Security and privacy concerns are identified as the main user barriers to adopting a digital health app (49). This is why this topic needs to be carefully addressed by app developers, which is also not sufficiently implemented in many popular apps in an academic setting (50). Based on the answers, some respondents have been aware of the security challenges in general. However, it seems a necessity to establish more knowledge in this area. The reporting of considerations and measures regarding security is under-represented in the systematic review and the survey. Only three review studies of the gave some information on this important issue. Data protection as part of the main topic security must be considered in compliance with national standards (8, 17). The survey showed that sports scientists have limited knowledge of how to address security and the corresponding security measures.

Findings on technology and dissemination showed that about half of the studies reported the underlying technology or were aware that the concept of dissemination needs to be defined. The findings of the survey showed basic knowledge, which is essential for the guidance of the app development process. Detailed answers to the questions for technology were not expected as in most cases the actual software development takes place with the support of experts or external companies. The reason dissemination is not primarily addressed might be influenced by the academic setting, where the motivation for app development is often based on specific research questions and less on the commercial success of the app. This is in line with the finding of the review that apps were limited to the study scope in five studies. However, it is crucial for commercial app success where on average 31% of the app budget is spent on marketing (51).

### 4.2. Research questions

The aim of this study is to give answers to the two research questions. Based on the answers to the five main topics, we conclude that there is a varying degree of software engineering knowledge in the sports science app development context (Q1). Furthermore, we conclude that the role of sports scientists within app development is not sufficiently defined (Q2).

Knowledge about software engineering principles is very important, for example, to guide and decide on a well-defined app development process that should allow quick response to user and stakeholder requirements and thus simplify and reduce the effort for implementation (20). Michie et al. (2017) reported recommendations for the development and evaluation of digital behavior change interventions. These recommendations have been derived from a two-day workshop with 42 participants. Relevant recommendations for app development outside of an academic sports science setting are to consider adopting methods from engineering and other data-intensive domains in the development cycle to achieve rapid and efficient development (17). This recommendation substantiates our opinion that it is important also for domain experts to have basic software engineering knowledge.

The survey showed that the background of the respondents is in the field of sports science. There is only a minority of respondents having a well-established background in computer science or information technology. This represents the constellation of sports science with several subdisciplines where sports information technology is not present in every institute in Germany. The findings under the section project information underline the missing experiences in terminology, e.g., the term product owner. Thus, self-perception of sports scientists is of a research assistant. We conclude that the role is not sufficiently understood. The findings of the systematic review showed that one-third of the publications are written by sports scientists involved in the app development process. In two other studies, the involvement of domain experts was explicitly described. The integration of domain experts within the whole development process is important as apps have the potential to harm, for example, when inappropriate advice is given (17).

We conclude that it is necessary to sharpen the understanding of roles in software engineering. Out of the findings, we neither derive conclusions for Q1 nor Q2. To the best of our knowledge, no similar study can be consulted to discuss the findings. Further research is needed to validate these findings and highlight the role and related success in app development.

### 4.3. Strengths and limitations

The strength of this study is the approach and the derivation of practical recommendations that can be put forth to promote successful app development and long-term maintenance to keep the app alive. Furthermore, the view on app development from the perspective of domain experts is valuable as often these are the driving forces or are asked for digital solutions for particular use cases.

The literature review does not reflect the actual number of apps in the field of sports science. The main publishers of digital health apps are start-up companies (9). It can be assumed that these start-ups do not focus on publishing their app development process, and thus, it is not possible to verify their procedures by involving domain experts. Thus, the generalizability of the findings is limited to the academic sports science setting. The limitations of the survey are sample selection, self-reported data collection, and the limited recruitment scope. Significantly, the small sample is a serious limitation.

Furthermore, the very important part of user experience during the development process has not been intensely considered in this study. User requirements and feedback are important inputs for app development and thus need to be considered during the entire development process (49). End users' needs are important for appropriate intervention, acceptance, and thus higher efficiency (50).

### 4.4. Recommendations

With a detailed discussion of the findings and considering the limitations, we derived five recommendations to improve the current situation in app development in the sports science setting. The results and discussion of the literature review and the survey do not confirm the initial research questions Q1 and Q2. To support project teams at the beginning of the development process to increase the success probability in terms of time, budget, and required functionality, we have derived five recommendations.

**Recommendation 1**: Use an agile development process.

Mata et al. (2015) highlighted the importance of incremental and agile development methodologies (20). This might also be beneficial in the context of defining the role of sports scientists. We recommend using an agile development process, for example, the SCRUM framework, which is used in most software projects (52). It provides an excellent basis for systematic development. SCRUM defines different roles and their responsibilities together with a couple of recurring events to systematize the tasks of the roles. We recommend that health, fitness, or sports scientists familiarize themselves with SCRUM before starting a project.

**Recommendation 2**: Health, fitness, or sports scientists should act as product owners or subject matter experts.

Based on the findings of this study, it is important to specify the role during app development. Health, fitness, or sports scientists should act as product owners. The healthcare domain is nontrivial, and misunderstandings between stakeholders and developers are common (20). Project initiation and domain knowledge are typically held by the scientists. That means details about how the app should work and which requirements are needed to be fulfilled have to be defined and articulated to the development team. Hereby, it is important to have a basic understanding of how good requirements are defined. Good requirements are necessary, appropriate, unambiguous, complete, singular, feasible, verifiable, correct, and conforming (53). Inappropriate requirements engineering is the major reason for failing projects (54). In the SCRUM framework, the product owner is responsible for defining and prioritizing requirements in the form of the so-called product backlog. Hereby, its main task is maximizing the value of the product (55). The product owner role is executed by a single person who takes over the responsibility and is accountable for all decisions. The product owner can be supported by several health, fitness, or sports scientists as subject matter experts.

**Recommendation 3**: Technical consultants should be included.

Software development is a complex endeavor and requires a lot of technical knowledge in addition to the process know-how (see recommendation 1). Even if this knowledge is available in some projects (see above), we recommend installing a technical consultant in each project that supports the project leader with respect to technical decisions. In particular, if development is outsourced to third parties, architectural decisions made by the development team shall be challenged for cost-efficient and sustainable development.

**Recommendation 4**: Outsource development to specialists.

Based on the survey results, we already see a trend toward outsourcing development to specialized companies or project partners with appropriate competencies. This is in line with affirming Q1 and concurrently a solution for the lack of knowledge of software engineering principles. The expectations from outsourcing are a faster time-to-market and a higher quality for the app, and thus a more efficient use of the budget. Finding a good and reliable development partner is however a challenging task that is not under full control of the project leader due to regulatory constraints.

**Recommendation 5:** Consider sustainability from the beginning.

Results from questions focusing on technology and dissemination indicated deficits in sustainability. From day one of the app development project, it shall be considered how sustainability can be guaranteed. Sustainability means that it is not sufficient to release the first version of the app. In particular, if a successful health, fitness, or sports app shall be realized, it is important to consider tasks after the first release, like ensuring that bugs are fixed and subsequent releases are planned. Also, it is necessary to cope with the continuously changing environment (e.g., new version operating systems) and to handle identified security vulnerabilities in used software components.

## 5. Conclusions

The digital health sector faces a rapidly changing technology landscape where most development is promoted by start-up companies (9, 17). It is known that domain experts need to be involved during the entire app development process (20) and these experts can be found in academic settings. This study aimed to give an overview of the current state of app development in an academic sports science setting. A systematic review and a survey in an exemplary academic setting were used as two sources of information to generate findings. One of the findings of our study is that it is quite helpful to establish a common understanding and basic know-how on software development and apply a defined software development approach. In line with this, it is important to define the role of the domain experts. The presented recommendations can be seen as best practice solutions to help academic project teams to define their starting point and support the implementation of potentially valuable solutions out of academia. Future research should focus on the reporting of app development and generalizability of these findings. To generalize the results, two directions should be considered. First, the survey should be extended to other research organizations. Second, the questions should be extended to other domains beyond sports science. Furthermore, additional aspects like user experience research that are crucial for app success need to be further elaborated.

## Data Availability

The original contributions presented in the study are included in the article/**Supplementary Material**; further inquiries can be directed to the corresponding author.
