# Supplementary figures and images for: App development in a sports science setting: A systematic review and lessons learned from an exemplary setting to generate recommendations for the app development process
Source: Front Sports Act Living. 2023 Jan 4;4:1012239. doi: 10.3389/fspor.2022.1012239 (PMC9845595; doi:10.3389/fspor.2022.1012239)

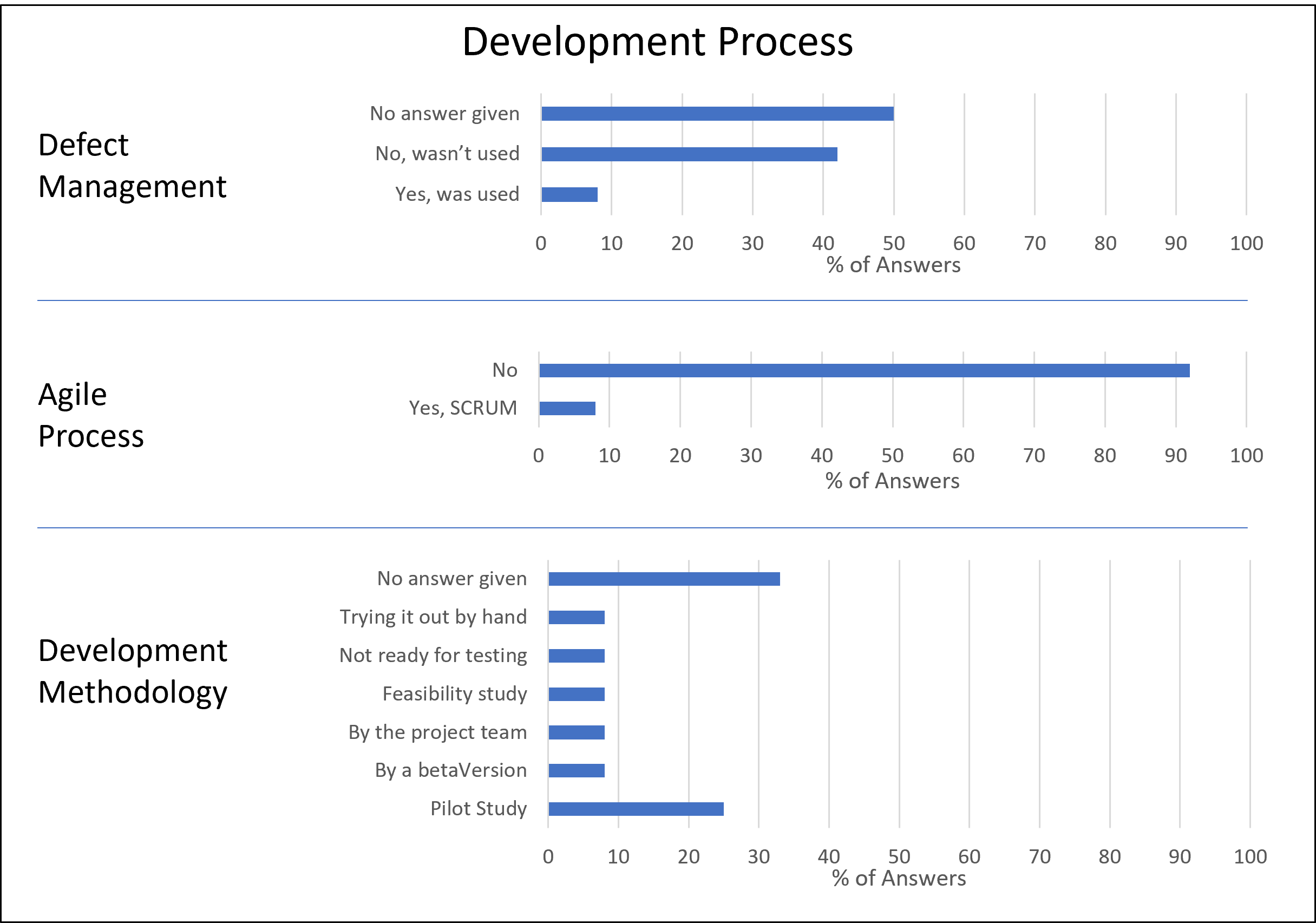

Supplement: Supplementary file 1 [file Image1.png]

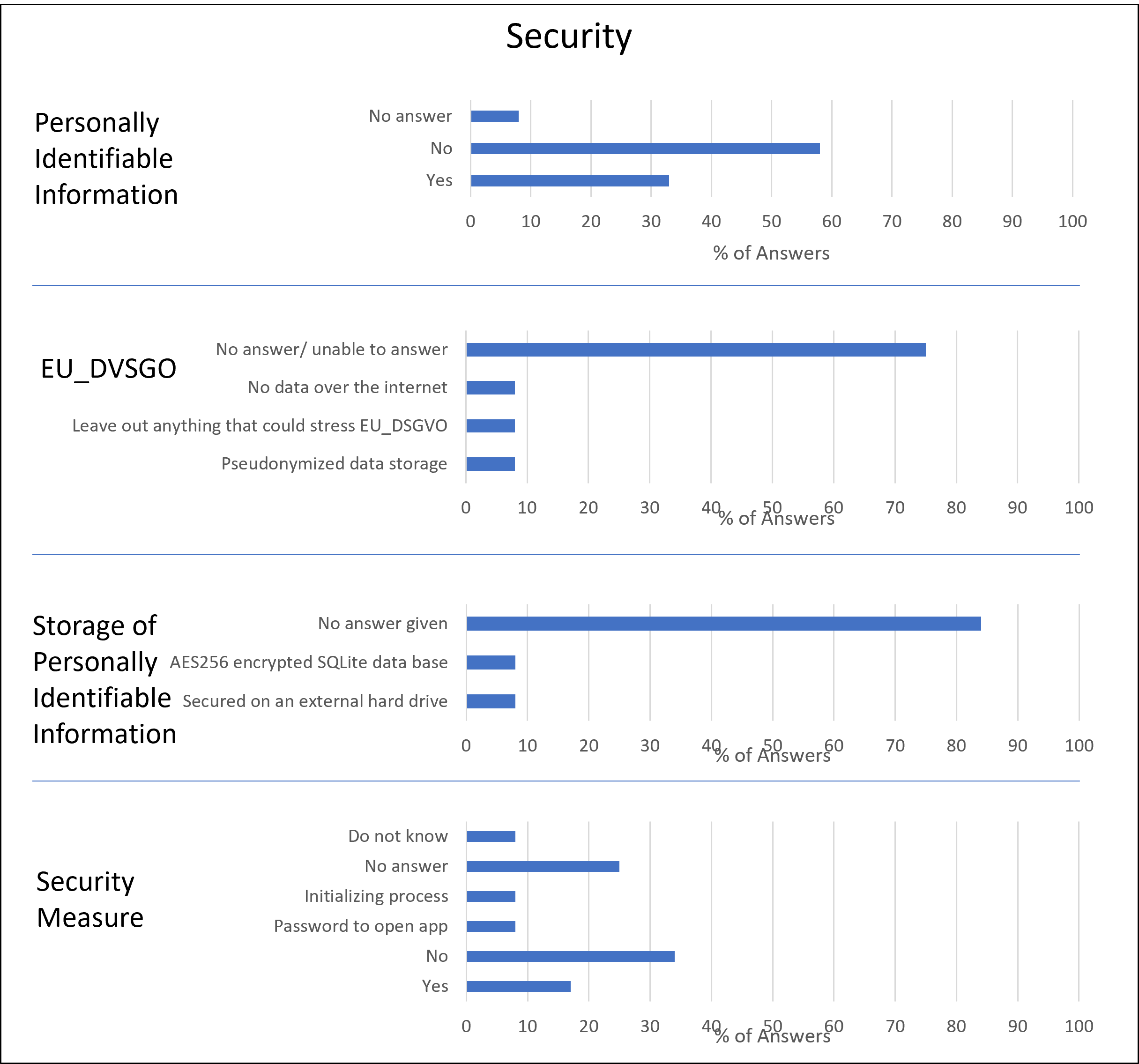

Supplement: Supplementary file 2 [file Image2.png]

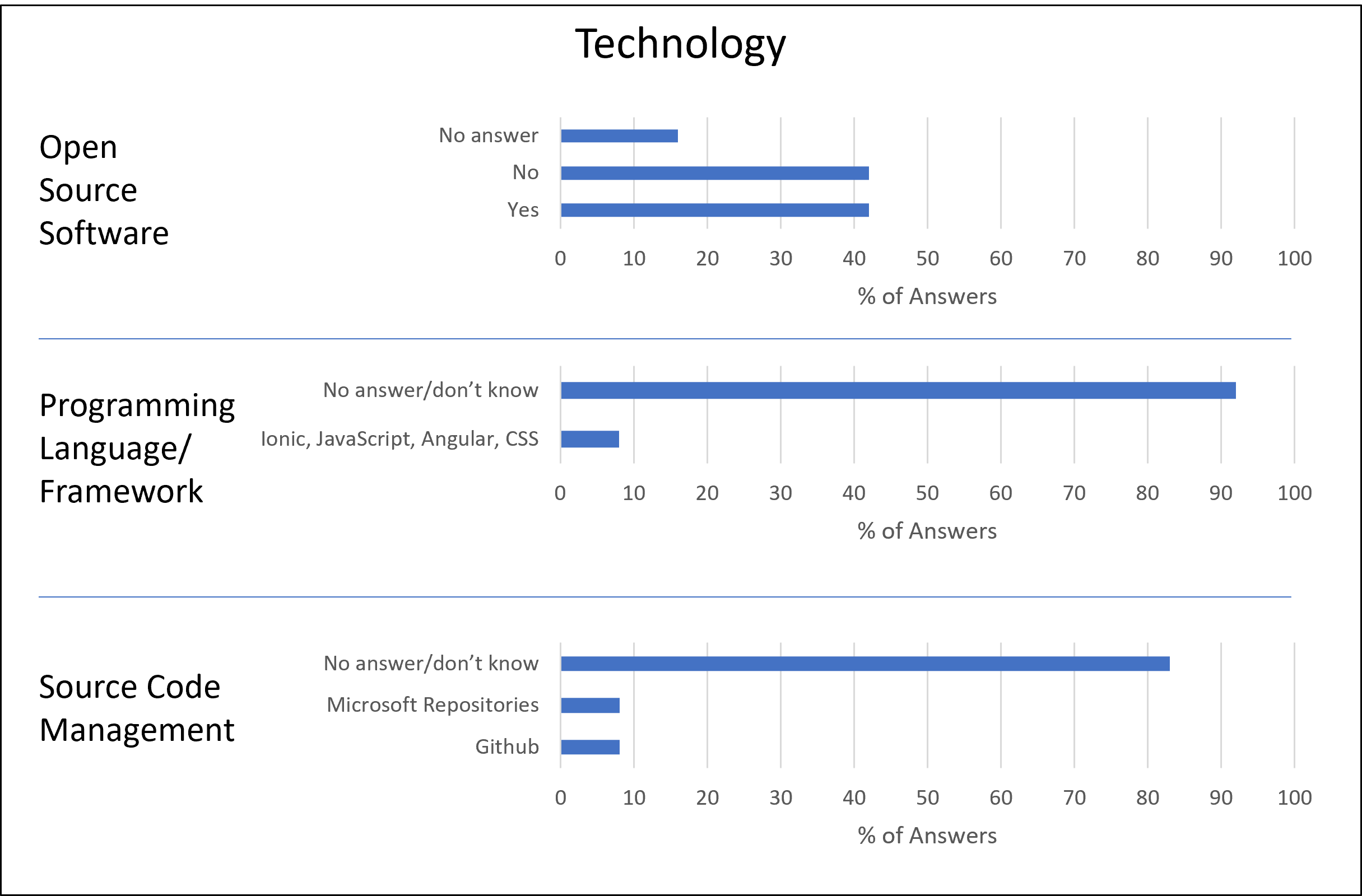

Supplement: Supplementary file 3 [file Image3.png]

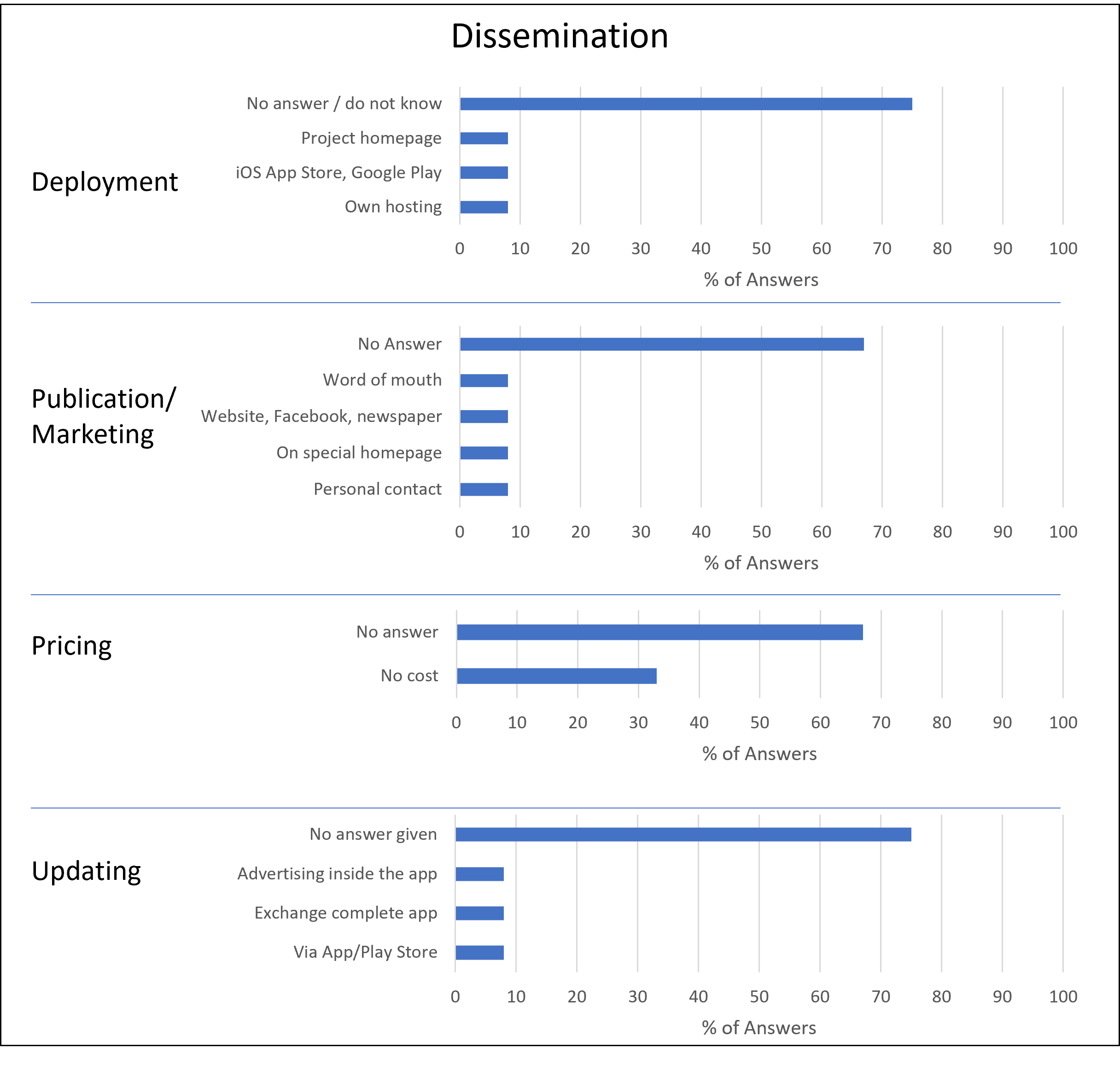

Supplement: Supplementary file 4 [file Image4.png]

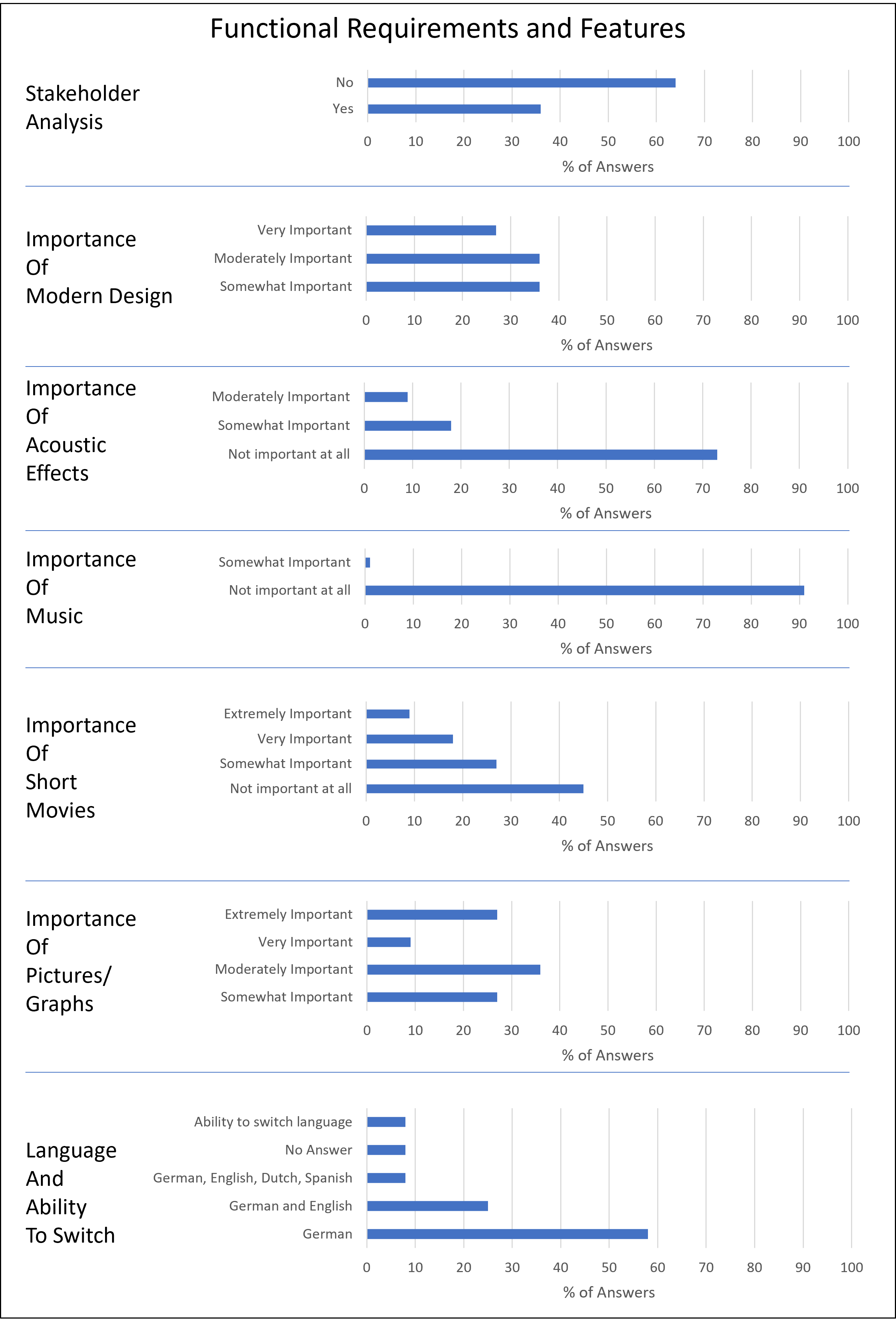

Supplement: Supplementary file 5 [file Image5.png]
